# UV-DDB as a Dynamic Regulator Linking Base Excision and Nucleotide Excision Repair via AAG Interaction

**DOI:** 10.3390/ijms27125521

**Published:** 2026-06-18

**Authors:** Jiwon Eom, Yubin Ko, Jeongwoo Choi, Soobin Yang, Su-Jin Kang, Seheon Kim, Yong Bhum Song, Soyeong An, Ja Yil Lee, Sunbok Jang

**Affiliations:** 1 Graduate School of Pharmaceutical Sciences, College of Pharmacy, Ewha Womans University, Seoul 03760, Republic of Korea; wonny529@ewha.ac.kr (J.E.); koyubin@ewha.ac.kr (Y.K.); wooyaa@ewhain.net (J.C.); binaida@ewha.ac.kr (S.Y.); 2 Graduate Program in Innovative Biomaterials Convergence, Ewha Womans University, Seoul 03760, Republic of Korea; 3 College of Pharmacy, Dongduk Women’s University, Seoul 02748, Republic of Korea; sjkang@dongduk.ac.kr; 4 Division of Research Center, Scripps Korea Antibody Institute, Chuncheon 24341, Republic of Korea; seheon6403@skai.or.kr (S.K.); songyb@skai.or.kr (Y.B.S.); 5 Department of Biological Sciences, Ulsan National Institute of Science and Technology, Ulsan 44919, Republic of Korea; sonya1004@unist.ac.kr (S.A.); biojayil@unist.ac.kr (J.Y.L.); 6 Graduate Program in Regulatory Science Convergence, Ewha Womans University, Seoul 03760, Republic of Korea

**Keywords:** UV-damaged DNA-binding protein (UV-DDB), alkyladenine DNA glycosylase (AAG), base excision repair (BER), nucleotide excision repair (NER), surface plasmon resonance (SPR), biolayer interferometry (BLI), AlphaFold3

## Abstract

Base excision repair (BER) and nucleotide excision repair (NER) are traditionally regarded as independent pathways; however, accumulating evidence indicates that ultraviolet (UV)-damaged DNA-binding protein (UV-DDB), a core NER factor, stimulates BER DNA glycosylases, including alkyladenine DNA glycosylase (AAG). Despite this functional link, the molecular basis of the UV-DDB/AAG interaction and its regulation by DNA remain unclear. This study investigated the direct interaction between AAG and UV-DDB using electrophoretic mobility shift assays (EMSA), surface plasmon resonance (SPR), biolayer interferometry (BLI) and AlphaFold3-based structural modeling under DNA-free and DNA-bound conditions. SPR analysis revealed that AAG and UV-DDB form a high-affinity complex in the absence of DNA ($K_{D}$ ≈ 17.5 nM), which is maintained but reduced approximately 2.6-fold upon binding to apurinic/apyrimidinic site (AP site)-containing dsDNA ($K_{D}$ ≈ 46.2 nM). BLI analysis independently confirmed this interaction under both DNA-free and DNA-bound conditions, with inter-platform differences consistent with previously reported BLI/SPR variability. EMSA showed UV-DDB-mediated ternary complex formation accompanied by redistribution of binary AAG/DNA species. AlphaFold3 modeling predicted that AAG associates with DDB1 in the DNA-free state, whereas under DNA-bound conditions, DDB2 recognizes the AP site while AAG repositions toward the lesion with multiple active site residues placed in close proximity. These findings support a model in which DNA binding acts as a molecular switch that reconfigures the UV-DDB/AAG interaction, potentially enabling UV-DDB to function as a recruitment platform that facilitates directional progression of AAG through the BER cycle, and providing a structural basis for coordinated integration of BER and NER.

## 1. Introduction

DNA damage is continuously induced by endogenous metabolic processes and exogenous environmental factors, which persistently threaten genomic stability. Reactive oxygen species, spontaneous hydrolytic reactions, and alkylating agents produce tens of thousands of base lesions per cell per day [1]. If unrepaired, these lesions interfere with transcription and replication and promote mutagenesis, cancer, and neurodegeneration [2,3,4]. To counter this damage, cells employ multiple DNA repair pathways, including BER and NER, which repair non-bulky and bulky DNA lesions, respectively [3,5].

BER removes small, non-helix-distorting base lesions through a pathway initiated by DNA glycosylases that generate AP site, subsequently processed by downstream repair enzymes [3,6]. Among these, alkyladenine DNA glycosylase (AAG) recognizes various damaged purines and initiates BER [7,8]. Notably, AAG strongly binds to AP site intermediates, which can limit dissociation and slow repair progression [7,9]. AAG dysregulation is linked to the accumulation of cytotoxic repair intermediates and neurodegeneration [10].

In contrast, NER targets helix-distorting DNA lesions such as UV-induced photoproducts [5]. The UV-damaged DNA-binding protein (UV-DDB) complex, composed of DDB1 and DDB2, functions as an initial damage sensor in global genome NER [11]. DDB2 directly recognizes damaged DNA, whereas DDB1 serves as a scaffold and participates in a CUL4A/RBX1 E3 ubiquitin ligase complex, promoting chromatin remodeling through histone ubiquitination [11,12,13].

Recent studies elucidate the role of UV-DDB beyond NER. It recognizes BER-associated lesions, including AP sites and oxidized bases, and stimulates multiple DNA glycosylases, including AAG, by promoting their dissociation from DNA [1,14,15]. These findings suggest that UV-DDB acts as a general damage sensor and regulator, highlighting a link between BER and NER pathways. In this context, DNA repair occurs within chromatin, where nucleosome organization restricts DNA accessibility [16,17]. UV-DDB facilitates repair by recognizing lesions in nucleosomal DNA and promoting local chromatin remodeling [1,18]. In addition, the DDB1-containing E3 ubiquitin ligase complex promotes repair through ubiquitin-mediated chromatin remodeling [12,13]. Despite these advances, most studies focus on DNA-bound conditions, emphasizing the role of UV-DDB in lesion recognition and glycosylase stimulation. However, little is known about how UV-DDB interacts with BER enzymes in the absence of DNA or how its presence influences these interactions at the molecular level. Since DNA repair proteins often undergo conformational and interaction changes upon DNA binding, UV-DDB/AAG interactions likely exist in distinct functional states.

Emerging evidence suggests that protein/protein interactions within DNA repair complexes are dynamically reconfigured by DNA, enabling coordinated transitions between damage recognition and processing [19]. In this context, the UV-DDB/AAG interaction suggests a DNA-dependent molecular switch, in which the complex adopts distinct configurations under DNA-free and DNA-bound conditions.

DNA-dependent conformational changes that remodel protein/protein interaction surfaces have been reported for several DNA repair enzymes, including PARP-1 [20,21], MutM [22], MutS [23,24], RPA, and APE1 [25,26,27]. These observations suggest that DNA binding acts as a molecular switch that coordinates sequential repair steps by remodeling the protein/protein interactions within repair complexes. By analogy, the UV-DDB/AAG interaction may be similarly regulated by DNA.

A previous study shows that UV-DDB physically interacts with AAG and enhances AAG-mediated lesion excision [7]. However, the binding affinity, kinetic properties, and structural basis of this interaction—particularly its modulation by DNA—remain unclear.

Therefore, this study investigated the UV-DDB/AAG interaction using biochemical and biophysical approaches, including EMSA, SPR, BLI and AlphaFold3-based structural modeling [28].

## 2. Results

### 2.1. Production and Validation of Functional UV-DDB and AAG Proteins

#### 2.1.1. Expression and Purification of UV-DDB and AAG Proteins

**Figure 1:**
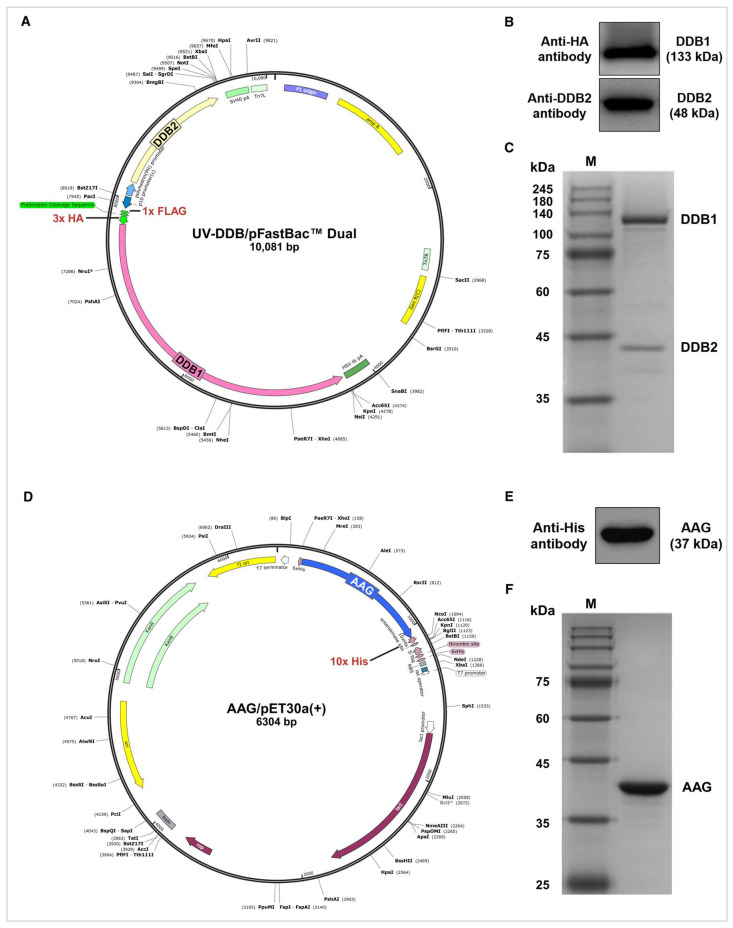
Production and validation of UV-DDB and AAG proteins. (**A**) Plasmid map of the baculovirus transfer vector encoding the human UV-DDB heterodimer (DDB1 and DDB2). The construct contains DDB1 and DDB2 coding sequences under dual promoters, with DDB1 fused to N-terminal FLAG and HA tags for purification and detection. Key regulatory elements, including the polyhedrin promoter, p10 promoter, and SV40 polyadenylation signal, are indicated. The PreScission protease cleavage site is located upstream of the tag sequence. Antibiotic resistance genes, replication origins, and selected restriction enzyme sites are annotated. Restriction sites marked with an asterisk (*) indicate enzymes whose activity may be affected by *dam* or *dcm* methylation of *Escherichia coli*-amplified DNA. (**B**) Western blot analysis of UV-DDB expression in Sf9 cells. DDB1 (~133 kDa) and DDB2 (~48 kDa) expression increased with baculovirus dose. DDB1 and DDB2 were detected using anti-HA and anti-DDB2 antibodies, respectively. (**C**) SEC purification of UV-DDB. SDS-PAGE analysis of collected fractions confirms UV-DDB purification. (**D**) Plasmid map of the AAG expression construct. The *AAG* gene was cloned into the pET30a(+) vector (*AAG*/pET30a(+), 6304 bp) under the T7 promoter for recombinant expression in *Escherichia coli*. The construct includes an N-terminal His_6_ tag for affinity purification. Key features, including the T7 terminator, lac operator, antibiotic resistance gene, and selected restriction sites, are indicated. (**E**) Western blot analysis of purified AAG following SEC. AAG was detected using an anti-His antibody, showing a single band at the expected molecular weight (~37 kDa), confirming protein identity and integrity. (**F**) SEC purification of AAG. SDS-PAGE of collected fractions confirms AAG purification.

UV-DDB was expressed using a baculovirus system, with the plasmid map showing the expression construct (Figure 1A). The vector encodes the human UV-DDB heterodimer (DDB1 and DDB2) under dual promoters, with DDB1 fused to N-terminal FLAG and HA tags for purification and detection. Expression and purification of UV-DDB were validated via Western blot using anti-HA and anti-DDB2 antibodies, detecting DDB1 (~133 kDa) and DDB2 (~48 kDa), respectively (Figure 1B). SDS-PAGE further confirmed UV-DDB purification, showing two bands corresponding to DDB1 and DDB2 (Figure 1C).

Recombinant AAG was produced using a pET30a(+) vector (Figure 1D), in which AAG is fused to an N-terminal His_6_ tag for affinity purification. Purified AAG was validated via Western blot using an anti-His antibody, showing a single band at the expected molecular weight (~37 kDa), confirming protein identity (Figure 1E). SDS-PAGE showed a prominent AAG band, indicating successful purification (Figure 1F).

#### 2.1.2. DNA-Binding Activity of UV-DDB and Its Modulation of AAG/DNA Interactions

**Figure 2:**
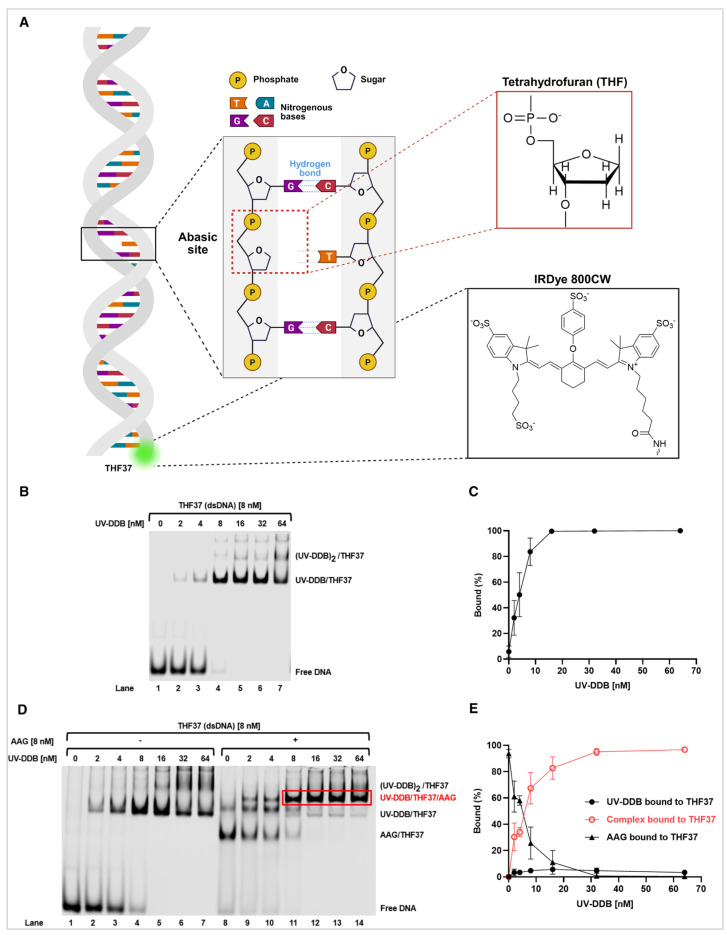
Electrophoretic analysis of UV-DDB binding to abasic DNA and modulation of AAG/DNA interactions. (**A**) Schematic of the IRDye 800-labeled double-stranded DNA substrate containing a THF AP site analog (THF37) used for EMSA. The DNA duplex is labeled at the 3′ end of the top strand with IRDye 800CW for fluorescence detection. The central region marks the abasic site, where THF replaces the natural base to mimic an AP site. Insets show the chemical structures of IRDye 800CW and THF. (**B**) EMSA showing UV-DDB binding to THF37 DNA. Increasing UV-DDB concentrations (0–64 nM) progressively shifted free DNA to slower-migrating DNA/protein complexes, indicating concentration-dependent binding. (**C**) Quantification of UV-DDB binding to THF37 DNA. The bound fraction increased with UV-DDB concentration, reaching near-saturation (~95%) at approximately 16 nM. (**D**) EMSA analysis of AAG/DNA and UV-DDB/DNA/AAG complex formation. In the absence or presence of AAG (8 nM), increasing UV-DDB concentrations promoted formation of a ternary complex, accompanied by redistribution of AAG/DNA complexes. (**E**) Quantification of binding species as a function of UV-DDB concentration. The ternary complex fraction increased with UV-DDB concentration, while the AAG/DNA complex decreased, indicating UV-DDB-mediated remodeling of AAG/DNA interactions.

To evaluate the DNA-binding activity of purified UV-DDB, EMSA were performed using AP site-containing dsDNA substrates. The substrate was a double-stranded oligonucleotide containing a tetrahydrofuran (THF) AP site analog and labeled at the 3′ terminus with IRDye 800CW for fluorescence detection (Figure 2A).

This analysis was prompted by our previous study showing cooperative interaction between AAG and UV-DDB during the removal of modified bases [7]. In the present study, we used independently purified AAG and UV-DDB complexes generated using a distinct expression and purification strategy. In addition, EMSA conditions and substrate design were optimized to enable a more controlled and quantitative assessment of DNA-binding behavior. Thus, these experiments represent a reconstituted system designed to validate and extend our previous findings under defined biochemical conditions.

Figure 2B shows that increasing UV-DDB concentrations progressively shifted the free DNA band to slower-migrating protein/DNA complexes, indicating concentration-dependent binding. At intermediate concentrations, most DNA was shifted, suggesting near-saturation of binding. At higher concentrations, an additional slower-migrating band appeared, consistent with higher-order UV-DDB/DNA complex formation. Quantitative analysis confirmed high-affinity binding, with >95% of DNA bound at approximately 16 nM (Figure 2C). These results demonstrate that purified UV-DDB retains strong DNA-binding activity and is functionally competent for downstream interaction and biophysical analyses.

Having established UV-DDB DNA-binding activity, we next examined its effect on AAG/DNA interactions using EMSA under comparable conditions. AAG alone formed a stable AAG/DNA complex, as indicated by a shift of free DNA to a slower-migrating band (Figure 2D). UV-DDB alone also bound DNA in a concentration-dependent manner, forming UV-DDB/DNA complexes.

Upon addition of UV-DDB to preformed AAG/DNA complexes, slower-migrating bands corresponding to ternary UV-DDB/DNA/AAG complexes were observed. The intensity of these bands increased with UV-DDB concentration, accompanied by a gradual reduction in the AAG/DNA complex, indicating a redistribution of DNA-bound species. Quantitative analysis revealed that the proportion of DNA in the ternary complex increased with UV-DDB concentration, while the AAG/DNA complex decreased (Figure 2E), supporting cooperative interaction between UV-DDB and AAG on abasic DNA.

### 2.2. Direct Interaction and DNA-Dependent Modulation of UV-DDB/AAG Interaction

#### 2.2.1. SPR Analysis Reveals High-Affinity UV-DDB/AAG Interaction

**Figure 3:**
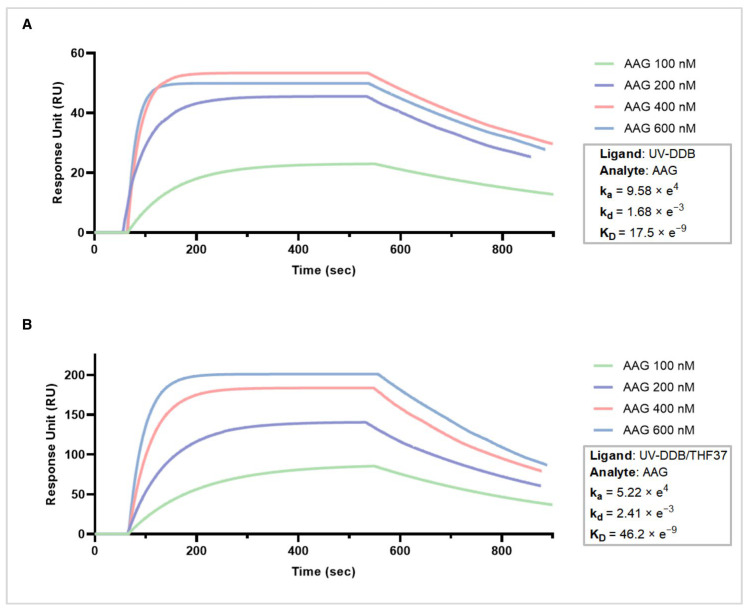
SPR analysis of AAG binding to UV-DDB in the absence and presence of DNA. (**A**) Sensorgrams showing concentration-dependent binding of AAG to immobilized UV-DDB in the absence of DNA. The response increases with AAG concentration and approaches saturation at higher concentrations. Data were globally fitted to a 1:1 binding model. (**B**) Sensorgrams showing concentration-dependent binding of AAG to immobilized UV-DDB/DNA complexes. The response increases with AAG concentration and approaches saturation, with minimal dissociation observed. Data were globally fitted to a 1:1 binding model.

To investigate the direct interaction between AAG and UV-DDB in the absence of DNA, SPR analysis was performed. AAG binding to immobilized UV-DDB produced a concentration-dependent increase in response units, indicating progressive association between the proteins (Figure 3A). At higher concentrations, the binding response approached saturation, suggesting near-complete occupancy of the binding surface.

Minimal signal loss was observed during dissociation, indicating slow dissociation and formation of a stable complex. Global fitting of sensorgrams to a 1:1 binding model yielded consistent kinetic parameters across all tested concentrations (100–600 nM), with a $k_{a}$ of 9.58 × 10^4^ M^−1^·s^−1^ and $k_{d}$ of 1.68 × 10^−3^ s^−1^, corresponding to a $K_{D}$ of approximately 17.5 nM. A representative overlay of the experimental sensorgrams and the corresponding globally fitted curves, together with the fitted kinetic parameters and their standard errors, is provided in Supplementary Figure S1A and Supplementary Table S1, and the kinetic parameters obtained from three independent replicate experiments (n = 3) are summarized in Supplementary Table S2A.

#### 2.2.2. AAG Binding to DNA-Bound UV-DDB Protein

SPR measurements showed that AAG binds to UV-DDB in the presence of AP site-containing dsDNA. Binding responses increased with AAG concentration, indicating efficient association with the UV-DDB/DNA complex (Figure 3B). At higher concentrations, the response approached saturation, indicating significant occupancy of available binding sites.

Minimal signal decay was observed during the dissociation phase, indicating slow complex dissociation and persistence. Kinetic analysis using a 1:1 binding model produced consistent parameters across all tested concentrations (100–600 nM), with a $k_{a}$ of 5.22 × 10^4^ M^−1^·s^−1^ and a $k_{d}$ of 2.41 × 10^−3^ s^−1^, yielding a $K_{D}$ of approximately 46.2 nM. These results show that the UV-DDB/AAG interaction persisted under DNA-bound conditions. A representative overlay of the experimental sensorgrams and the corresponding globally fitted curves, together with the fitted kinetic parameters and their standard errors, is provided in Supplementary Figure S1A and Supplementary Table S1, and the kinetic parameters obtained from three independent replicate experiments (n = 3) are summarized in Supplementary Table S2B.

#### 2.2.3. BLI Analysis Confirms High-Affinity UV-DDB/AAG Interaction and Its DNA-Dependent Modulation

To further characterize the direct interaction between AAG and UV-DDB under both DNA-free and DNA-bound conditions, BLI analysis was performed. AAG binding to immobilized UV-DDB produced concentration-dependent increases in binding signal during the association phase, indicating progressive complex formation between the two proteins. At higher AAG concentrations, the binding response approached saturation, consistent with near-complete occupancy of available binding sites. During the dissociation phase, only limited signal decay was observed, indicating slow dissociation kinetics and formation of a stable UV-DDB/AAG complex. Global fitting of the BLI sensorgrams to a 1:1 binding model yielded $K_{D}$ of approximately 84.1 nM (R^2^ = 0.9892) (Supplementary Figure S2A). These results confirm the formation of a direct and stable UV-DDB/AAG complex in the absence of DNA, and further support the high-affinity interaction profile observed by SPR.

To further assess whether DNA engagement by AAG modulates this interaction, BLI analysis was additionally performed using AAG pre-incubated with THF37 dsDNA. Binding signals again increased in a concentration-dependent manner, indicating progressive association of the AAG/DNA complex with immobilized UV-DDB. At higher concentrations, the response approached saturation, consistent with substantial occupancy of available binding sites, and only limited signal decay was observed during the dissociation phase, indicating persistent slow dissociation kinetics. Global fitting of the sensorgrams to a 1:1 binding model yielded a $K_{D}$ of approximately 128.9 nM (R^2^ = 0.9375) (Supplementary Figure S2B). Compared to the DNA-free condition ($K_{D}$ ≈ 84.1 nM), binding affinity was moderately reduced in the presence of DNA; nevertheless, the interaction remained stable, demonstrating that UV-DDB/AAG complex formation is maintained even when AAG is pre-engaged with a DNA substrate.

### 2.3. AlphaFold3-Based Structural Prediction of the UV-DDB/AAG Complex

#### 2.3.1. Predicted UV-DDB/AAG Interaction in the Absence of DNA

**Figure 4:**
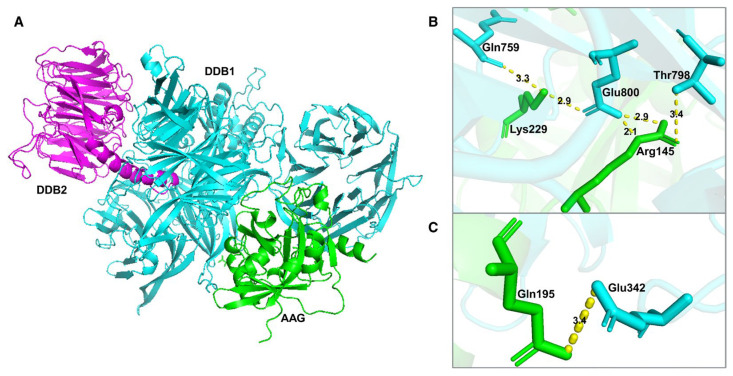
AlphaFold3-predicted structure of the UV-DDB/AAG complex in the absence of DNA. (**A**) Overall architecture of the predicted UV-DDB/AAG complex under DNA-free conditions. DDB2 is shown in magenta, DDB1 in cyan, and AAG in green. (**B**) Close-up view of the predicted DDB1/AAG interface at the primary contact site, where multiple DDB1 residues simultaneously engage AAG. (**C**) Close-up view of an additional contact at the predicted DDB1/AAG interface. Distances between selected atom pairs are indicated in ångströms (Å).

To elucidate the structural basis of UV-DDB/AAG complex formation in the absence of DNA, AlphaFold3 was used to predict the three-dimensional architecture of the complex. The predicted structure indicates a direct protein/protein interaction between AAG and the DDB1 subunit of UV-DDB (Figure 4A). Interface analysis identified six putative contacts between three AAG residues (Arg145, Gln195, and Lys229) and four DDB1 residues (Glu800, Thr798, Glu342, and Gln759) through hydrogen bonds and salt bridges (Figure 4B,C; Table 1). The closest predicted contacts were observed between AAG Arg145 and DDB1 Glu800, with inter-atomic distances of 2.1 Å (NE–OE2) and 2.9 Å (NH2–OE2). Additional contacts were identified between AAG Arg145 and DDB1 Thr798 (NH1–OG1: 3.4 Å), AAG Gln195 and DDB1 Glu342 (NE2–OE2: 3.4 Å), AAG Lys229 and DDB1 Gln759 (NZ–O: 3.3 Å), and AAG Lys229 and DDB1 Glu800 (NZ–OE1: 2.9 Å). No hydrophobic or aromatic interactions were identified at this interface.

#### 2.3.2. In Silico Alanine Substitution Modeling of Predicted DDB1/AAG Interface Residues

To evaluate the functional significance of the predicted interface residues, three alanine substitution variants were modeled using AlphaFold3: DDB1(Glu800Ala), AAG(Arg145Ala), and AAG(Lys229Ala). These residues were selected because each is predicted to simultaneously contact two or more distinct partner residues in the wild-type complex: DDB1 Glu800 contacts both AAG Arg145 and Lys229, AAG Arg145 contacts both DDB1 Glu800 and Thr798, and AAG Lys229 contacts both DDB1 Gln759 and Glu800. The distances of all six wild-type contacts were remeasured in each mutant model and compared with wild-type values.

In the DDB1(Glu800Ala) model, all contacts involving Glu800 were abolished, with distances increasing to 38.5–40.4 Å. The Gln195–Glu342 distance, which does not directly involve the substituted residue, also increased markedly from 3.4 Å to 43.5 Å. In the AAG(Arg145Ala) model, contacts between Arg145 and Glu800 and between Arg145 and Thr798 were lost, with distances increasing to 41.4–42.3 Å, while the Gln195–Glu342 distance increased to 13.0 Å. In the AAG(Lys229Ala) model, contacts between Lys229 and Gln759 and between Lys229 and Glu800 were disrupted, with distances increasing to 25.1–30.0 Å, and the Gln195–Glu342 distance increased to 18.7 Å (Table 2).

#### 2.3.3. Structural Prediction Under DNA-Bound Conditions

**Figure 5:**
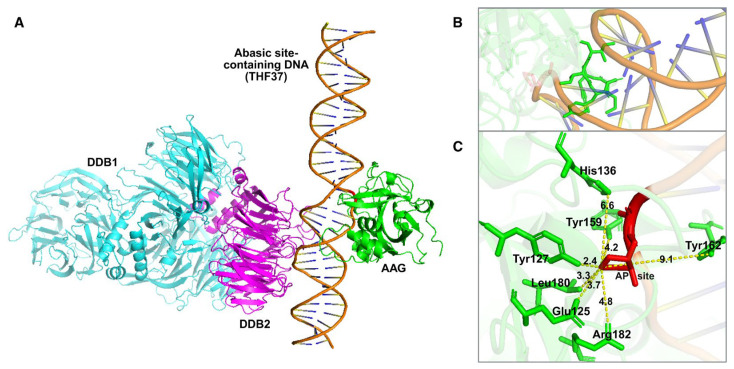
AlphaFold3-predicted structure of the UV-DDB/DNA/AAG ternary complex. (**A**) Overall architecture of the predicted ternary complex incorporating AP site-containing dsDNA (THF37). DDB1 is shown in cyan, DDB2 in magenta, AAG in green, and DNA in orange. AAG is positioned at the lesion site, while DDB2 is located adjacent to the DNA. (**B**) View showing insertion of the AAG β-hairpin into the minor groove of the AP site-containing dsDNA. (**C**) Close-up view of the AAG/DNA interface. Multiple AAG active site residues are positioned in proximity to the AP site C1′ atom. Distances between selected atom pairs are indicated in ångströms (Å).

To characterize the structural basis of the UV-DDB/AAG interaction under DNA-bound conditions, AlphaFold3 was used to predict the three-dimensional structure of the AAG/UV-DDB/DNA ternary complex incorporating a double-stranded AP site-containing dsDNA substrate (THF37). In the predicted ternary complex, AAG was positioned at the lesion site while DDB2 was located adjacent to the DNA (Figure 5A). The AAG β-hairpin was observed to insert into the minor groove of the AP site-containing dsDNA (Figure 5B). Multiple AAG active site residues were positioned in proximity to the AP site C1′ atom (Figure 5C; Table 3).

Tyr127 (OH–C1′ distance: 2.4 Å) and Glu125 (OE2–C1′ distance: 3.7 Å) were the closest predicted contacts to the AP site C1′ atom. Leu180 (CD2–C1′ distance: 3.3 Å), Tyr159 (OH–C1′ distance: 4.2 Å), Arg182 (NH1–C1′ distance: 4.8 Å), and His136 (NE2–C1′ distance: 6.6 Å) were additionally identified within 6.6 Å of the AP site. Tyr162 (OH–C1′ distance: 9.1 Å) was the most distal residue identified within this range.

## 3. Discussion

The present findings suggest that AAG and UV-DDB form a direct protein/protein complex in a DNA-independent manner, which is retained in the presence of AP site-containing dsDNA, albeit with an approximately 2.6-fold reduction in affinity [7]. Convergent evidence from EMSA, SPR, and BLI analyses suggests that UV-DDB promotes ternary complex formation with AAG on damaged DNA substrates, accompanied by redistribution of binary AAG/DNA species [7,14]. In parallel, AlphaFold3-based structural modeling is consistent with DNA-dependent repositioning of AAG from DDB1 toward lesion-proximal regions near DDB2. Collectively, these findings are consistent with a DNA-dependent molecular switch that may regulate the architecture and functional state of the UV-DDB/AAG complex [7].

A systematic discrepancy in $K_{D}$ values between BLI and SPR was observed under both DNA-absent and DNA-present conditions, with BLI-derived values approximately 4.8-fold higher in the DNA-free condition and 2.8-fold higher in the DNA-present condition relative to SPR. BLI and SPR are both real-time, label-free optical biosensor technologies, but use different fluidic designs [34]. The observed difference is consistent with previously reported challenges inherent to BLI, including analyte rebinding at higher ligand surface densities and mass transport limitation [34]. Consistent with existing literature, BLI-derived $K_{D}$ values have been reported to be 1.2- to 18-fold higher than SPR-derived values across a wide range of molecular interactions [35,36]. The discrepancy observed in the present study falls within this range and is therefore consistent with previously documented inter-platform variability. Notably, the difference was narrower under DNA-present conditions than under DNA-absent conditions, which may suggest that DNA binding alters the molecular size or hydrodynamic properties of the complex in a manner that partially mitigates differential detection sensitivity between the two platforms [34,37].

SPR kinetic data demonstrate that the UV-DDB/AAG complex adopts two distinct binding states depending on the presence of DNA. In the absence of DNA, AAG binds UV-DDB with high affinity and slow dissociation kinetics, and AlphaFold3 structural modeling predicts that AAG primarily contacts the DDB1 subunit in this state. This suggests that AAG may exist in a pre-assembled complex with UV-DDB prior to lesion recognition. In the presence of DNA, binding affinity is reduced approximately 2.6-fold, and the AlphaFold3 model predicts repositioning of AAG from the DDB1-dominated interface toward the DDB2/DNA interface. Together, these observations indicate that DNA binding acts not to disrupt the UV-DDB/AAG interaction, but to reconfigure its geometry and contact surface.

These two observations are reconcilable within the well-characterized bipartite kinetic behavior of AAG on DNA [38]. During lesion search, AAG occupies a low-affinity, non-committal scanning state that is intrinsically non-productive in the absence of targeting cues; once it commits to a substrate or, following catalysis, to an AP site product, AAG transitions into a high-affinity, slowly dissociating configuration that sequesters the enzyme and constitutes a recognized rate-limiting step in BER [38]. The present data suggest that UV-DDB may act at both ends of this catalytic cycle. In the pre-engagement phase, the qualitative shift from non-saturable BLI association to saturable, kinetically resolved SPR binding in the presence of UV-DDB is consistent with UV-DDB displacing AAG from its non-productive scanning mode into a defined, lesion-proximal configuration [7]. In the post-catalytic phase, the reduced affinity of the ternary complex and the redistribution of binary AAG/DNA species observed by EMSA and SPR are consistent with the possibility that UV-DDB may modulate AAG retention on AP site-containing dsDNA [7]. It should be noted, however, that the THF AP site analog used in the present study is a non-hydrolyzable mimic rather than a genuine enzymatic product; accordingly, whether UV-DDB actively facilitates release of catalytically product-trapped AAG from authentic AP site intermediates remains to be directly demonstrated. The present data are nonetheless consistent with the hypothesis that UV-DDB engagement reduces AAG occupancy at AP sites, a function that, if confirmed with native substrates, would be mechanistically analogous to the stimulatory effect on AAG-mediated lesion excision reported previously [7,14]. Rather than representing mechanistically unrelated activities, these observations may reflect complementary facets of a single allosteric mechanism in which the DNA-dependent conformational state of UV-DDB remodels the AAG/DNA interface to favor directional progression from non-productive scanning to lesion-proximal engagement. Whether this mechanism extends to the post-catalytic phase—specifically, whether UV-DDB facilitates AAG release following base excision—represents a functionally important question that warrants direct experimental investigation. This bidirectional regulatory function is conceptually analogous to that proposed for APE1 [9], which both accepts handoff of the AP site product from AAG and stimulates its dissociation [39]; UV-DDB may fulfill a complementary role at the entry point of this cycle [1,14].

AlphaFold3-predicted structures offer a potential mechanistic basis for this transition. In the DNA-free model, AAG is predicted to engage a previously uncharacterized interface on DDB1, distinct from canonical DDB1/DCAF interactions, consistent with the established role of DDB1 as a protein/protein interaction scaffold within the UV-DDB complex [11,40]. In the DNA-bound ternary complex, DDB2 is predicted to serve as the primary DNA-engaging subunit, while AAG is predicted to be positioned proximal to the lesion in a pre-engagement configuration. The DDB2 β-hairpin loop is inserted into the DNA minor groove at the AP site; however, because the AP site represents a position from which the damaged base has already been excised, this insertion does not engage an intact nucleotide and therefore cannot fulfill the nucleotide-flipping function described for intact lesions [11]. Tyr162, the intercalating residue of AAG known to stabilize the extrahelical conformation of the damaged base, is located at a distance substantially exceeding that observed in binary AAG/DNA crystal structures upon full intercalation [29,33]. This is interpreted to reflect a structural constraint imposed by the absence of a flippable base at the AP site, whereby AAG is oriented toward the lesion but has not adopted a fully intercalated catalytic conformation. Notably, residues within DDB2 known to contact DNA in binary complexes appear to be repositioned in the ternary model, raising the possibility that AAG co-binding may induce structural rearrangement within the UV-DDB complex [11].

The in silico alanine substitution analysis identifies DDB1 Glu800 as a central anchor residue at the predicted DDB1/AAG interface. Among the three substitution models, DDB1(Glu800Ala) produced the most pronounced structural perturbation: not only were all direct Glu800-mediated contacts abolished, but the Gln195–Glu342 distance—a contact not directly involving the substituted residue—increased from 3.4 Å to 43.5 Å, indicating a large-scale displacement of the entire AAG binding region from the DDB1 surface rather than loss of an isolated contact. This suggests that Glu800 functions as the primary organizer of the DDB1/AAG interface, and its removal destabilizes the overall binding geometry. In contrast, the AAG(Arg145Ala) and AAG(Lys229Ala) models showed more moderate increases in the Gln195–Glu342 distance (13.0 Å and 18.7 Å, respectively), indicating partial rather than wholesale displacement of AAG from the interface upon single-residue substitution. Taken together, these results support the functional importance of Glu800 as a disproportionate contributor to interface organization, and identify Arg145 and Lys229 as cooperative binding partners whose simultaneous engagement is required for stable complex formation. Experimental validation of these predicted interface residues is planned as a key objective of future follow-up studies; generation and purification of the corresponding mutant proteins, followed by multi-modal characterization encompassing SPR-based binding affinity analysis, crosslinking mass spectrometry (XL-MS), and cryo-electron microscopy (Cryo-EM), is expected to provide direct structural and biochemical confirmation of their respective contributions to the DDB1/AAG interaction interface.

This DNA-dependent structural organization is conceptually analogous to the transition observed in the NER endonuclease XPF/ERCC1, in which DNA junction engagement releases autoinhibition and enables catalytic activation [41]. In both systems, the presence of DNA drives a shift from a protein/protein interaction-dominated state toward a DNA-centered, functionally active configuration, illustrating a structural paradigm for coordinating sequential steps in DNA repair [41].

These findings further suggest a broader role for UV-DDB as a BER co-factor [1]. Beyond its established function in NER, UV-DDB has been shown to recognize diverse DNA lesions and stimulate multiple DNA glycosylases, including OGG1, MUTYH, and SMUG1 [1,14]. The pre-recruitment complex identified here may therefore represent a general mechanism through which UV-DDB positions BER enzymes for rapid lesion engagement [1,42]. In this context, the DNA-dependent molecular switch provides a structural and kinetic framework for integrating lesion recognition with downstream repair processing [7].

The biological relevance of this mechanism is supported by cellular and in vivo observations. Loss-of-function mutations in DDB2, as observed in xeroderma pigmentosum group E, result in defective repair and increased cancer susceptibility, highlighting the importance of stable repair complex formation. Similarly, AAG deficiency produces tissue-specific phenotypes, indicating that tight regulation of BER initiation is physiologically critical [1,7,43]. The UV-DDB/AAG interaction described here may therefore represent a regulatory checkpoint that coordinates repair efficiency across different cellular contexts [1,7,14].

From a therapeutic perspective, the in silico alanine substitution analysis performed in this study provides a structural basis for inhibitor design targeting the DDB1/AAG axis. Interaction hotspots—a small subset of interface residues that contribute disproportionately to binding free energy—represent attractive targets for small-molecule or peptidomimetic inhibitors, as their disruption is expected to destabilize the protein/protein complex [44]. As the sole DDB1 residue predicted to simultaneously engage two AAG contact partners, Arg145 and Lys229, Glu800 is proposed as a priority target for experimental validation through charge-reversal mutagenesis and binding affinity measurements.

Although the AlphaFold3 models presented here are based solely on computational prediction, they are internally consistent with the biophysical data obtained by EMSA, SPR, and BLI, and provide a mechanistically interpretable structural framework for the observed DNA-dependent modulation of the UV-DDB/AAG interaction [28,44]. The present study focuses on in vitro biochemical and biophysical characterization using purified components; future studies employing cellular systems, site-directed mutagenesis, cryo-electron microscopy, and crosslinking mass spectrometry will be important to further validate and refine the structural and functional basis of the UV-DDB/AAG interaction in physiologically relevant contexts [7,14]. These future directions represent a natural extension of the current findings rather than a prerequisite for establishing the core mechanistic model.

In summary, this study provides evidence for a DNA-dependent molecular switch governing the UV-DDB/AAG interaction and offers a mechanistic framework for understanding how BER and NER pathways may be functionally integrated through dynamic reorganization of repair complexes [1,7,14].

## 4. Materials and Methods

### 4.1. UV-DDB Protein Preparation

The construct of the pFastBac™ Dual Expression Vector encoding the human UV-DDB heterodimer (DDB1 and DDB2) was used without modification for recombinant protein expression in insect cells. Human DDB1 and DDB2 coding sequences were cloned into this vector, and DDB1 was engineered with N-terminal FLAG and HA tags to facilitate affinity purification.

Recombinant bacmid DNA was amplified in DH10Bac competent cells and purified using a NucleoBond Xtra Midi kit (740410.50, Macherey-Nagel, Düren, Germany). Purified bacmid DNA was transfected into Sf9 cells using Cellfectin^®^ II reagent (10362-100, Gibco, Thermo Fisher Scientific, Waltham, MA, USA). Sf9 insect cells (11496015, Gibco, Thermo Fisher Scientific, Waltham, MA, USA) were maintained in serum-free Sf-900™ II SFM (10902088, Gibco, Thermo Fisher Scientific, Waltham, MA, USA) and grown at 27 °C under continuous orbital agitation (125–130 rpm), maintaining a cell density of 2 × 10^6^ and 4 × 10^6^ cells/mL and viability >95%. Viral stocks were subsequently amplified through sequential passages (P0–P3). For large-scale protein production, Sf9 cultures were infected with the P3 virus and incubated at 27 °C until cell viability decreased to 60–70%.

Harvested cells were disrupted in lysis buffer containing 50 mM potassium phosphate (pH 8.0), 500 mM NaCl, 1 mM EDTA, 0.25 mM TCEP, 10% glycerol, and an EDTA-free protease inhibitor cocktail. After clarification and filtration (0.45 μm filter, SE1M003M00, Merck, Darmstadt, Germany), the lysate underwent affinity purification using ANTI-FLAG^®^ M2 resin (Sigma-Aldrich, Burlington, MA, USA). The resin was preconditioned with glycine-HCl (pH 3.5) and equilibrated in PBS before sample loading.

After thorough washing, bound proteins were eluted using FLAG peptide (0.1 mg/mL, F3290, Sigma-Aldrich, Burlington, MA, USA). The eluted fraction was concentrated with a Vivaspin 20 device (VS2002, Sartorius, Göttingen, Germany) and further purified using size-exclusion chromatography (SEC) on a HiPrep 16/60 Sephacryl S-200 HR column (GE Healthcare, Marlborough, MA, USA), equilibrated in buffer containing 20 mM HEPES (pH 7.8), 200 mM NaCl, 1 mM EDTA, 0.01% Triton X-100, 1 mM DTT, and 10% glycerol. The purified protein was concentrated, snap-frozen in liquid nitrogen, and stored at −80 °C.

### 4.2. AAG Protein Preparation

For AAG production, Rosetta (DE3) *E. coli* cells were transformed with a pET30a(+)-*AAG* expression plasmid. Transformed cells were cultured in LB medium containing kanamycin (50 μg/mL) and chloramphenicol (35 μg/mL) and grown at 37 °C to mid-log phase (OD_600_ ≈ 0.6–0.7). Protein expression was induced with IPTG (0.25 mM final concentration), followed by incubation at 18 °C for 18–20 h.

Cell pellets were resuspended in lysis buffer (50 mM potassium phosphate, pH 7.5, 500 mM NaCl, 25 mM imidazole, 10% glycerol, 0.5 mM PMSF) and lysed using sonication. After centrifugation, the supernatant was loaded onto a HisPur Ni-NTA column (88222, Thermo Fisher Scientific, Waltham, MA, USA). Bound AAG was eluted using an imidazole gradient (25−300 mM).

Eluted fractions were pooled, concentrated, and further purified via SEC on a HiPrep 16/60 Sephacryl S-200 HR column, equilibrated with buffer containing 20 mM HEPES (pH 8.0), 200 mM KCl, 1 mM EDTA, 1 mM DTT, and 10% glycerol. The final protein was concentrated, flash-frozen, and stored at −80 °C.

### 4.3. Protein Quantification (BCA Assay)

Protein concentrations were measured using a BCA assay kit (Thermo Fisher Scientific, Waltham, MA, USA). Absorbance at 562 nm was recorded with a multifunctional microplate reader (Spark, Tecan Group Ltd., Männedorf, Switzerland; NFEC-2024-09-299675) at the Ewha Drug Development Research Core Center.

### 4.4. Western Blotting

UV-DDB or AAG was loaded onto polyacrylamide gels and separated in running buffer at 100 V using a Mini-PROTEAN Tetra Cell (1658001, BIO-RAD, Hercules, CA, USA). After separation, proteins were transferred onto a PVDF membrane (IPVH00010, Merck) using a Mini Trans-Blot Cell with transfer buffer containing 20% methanol at 300 mA for 1 h 30 min. The membrane was blocked for 1 h in PBS-T containing 5% skim milk, followed by overnight incubation at 4 °C with primary antibodies: anti-HA (1:10,000, #26183, Thermo Fisher Scientific, Waltham, MA, USA), anti-DDB2 (1:200, sc81246, Santa Cruz Biotechnology, Dallas, TX, USA), or anti-His (1:2000, a190-114A, Fortis Life Sciences, Waltham, MA, USA). After four washes with PBS-T containing 5% skim milk (15 min each), the membrane was incubated for 1 h at room temperature with HRP-conjugated secondary antibodies: anti-mouse IgG (1:2000, ab97023, Abcam, Cambridge, UK) or anti-rabbit IgG (1:2000, ab205718, Abcam, Cambridge, UK), followed by four additional washes and detection using ECL substrate (32209, Thermo Fisher Scientific, Waltham, MA, USA). Using a Western blot imaging system (GBOX CHEMI XX9, Syngene, Cambridge, UK; NFEC-2022-12-283636) at Ewha Drug Development Research Core Center, protein bands were visualized, with exposure times optimized to maintain linear signal detection and prevent saturation.

### 4.5. DNA Substrate Preparation

Oligonucleotides were synthesized and HPLC-purified by Integrated DNA Technologies (IDT, Coralville, IA, USA). The following sequences (X = THF) were used:

Top: 5′-CCG AGT CAT TCC TGC AGC GXG TCC ATG GGA GTC AAA T [IRDye 800CW]-3′
Bottom: 5′-ATT TGA CTC CCA TGG ACT CGC TGC AGG AAT GAC TCG G-3′

Double-stranded DNA substrates containing a THF abasic site analog were generated by annealing complementary oligonucleotides. Equimolar strands were mixed in buffer comprising 10 mM Tris-HCl (pH 8.0) and 100 mM KCl, heated to 95 °C for 5 min, and slowly cooled to room temperature to facilitate duplex formation. The IRDye 800CW fluorophore was conjugated to the 3′ terminus of the top strand. This labeled substrate was used for electrophoretic mobility shift assay (EMSA) and biolayer interferometry (BLI) experiments.

For surface plasmon resonance (SPR) experiments, a distinct 40-mer duplex substrate was employed due to the requirement for surface immobilization via biotin–streptavidin capture on the sensor chip. Oligonucleotides were synthesized by Bioneer (Daejeon, Republic of Korea). The following sequences (X = THF) were used:

Top: 5′-[NH2] GTG ATT GCG TTG CGC TCA XTG TAT TCC ACA CAA CAT GCA T-3′
Bottom: 5′-[Biotin] ATG CAT GTT GTG TGG AAT ACA GTG AGC GCA ACG CAA TCA C-3′

The biotin moiety conjugated to the 5′ terminus of the bottom strand enabled stable capture of the duplex onto a streptavidin-coated SPR sensor chip, which is a prerequisite for SPR-based binding analysis, as the analyte (protein) flows over the chip surface in solution while the DNA ligand is immobilized. An amino (NH2) modification at the 5′ terminus of the top strand was incorporated to allow directional orientation of the duplex on the chip surface. The 40-mer length was selected to provide sufficient spacing from the chip surface and to minimize steric interference with protein binding to the internal THF abasic site. Duplex formation was performed using the same annealing procedure described above: equimolar complementary strands were mixed in 10 mM Tris-HCl (pH 8.0) and 100 mM KCl, heated to 95 °C for 5 min, and gradually cooled to room temperature.

### 4.6. Electrophoretic Mobility Shift Assay (EMSA)

To investigate DNA-dependent complex formation, AAG (8 nM) was incubated with THF37 DNA (8 nM) in reaction buffer (20 mM HEPES, pH 7.5, 150 mM NaCl, 2 mM DTT, 0.5 mg/mL BSA, and 5% glycerol) for 10 min at room temperature to assemble AAG/DNA complexes. UV-DDB was subsequently added at increasing concentrations (0–64 nM) and incubated for 30 min, resulting in a final reaction volume of 10 μL. Samples were loaded onto pre-run 5% native polyacrylamide gels (37.5:1 acrylamide:bisacrylamide) and electrophoresed at 100 V for 50 min at 4 °C in 0.5× TBE buffer. An Odyssey F imaging system was used to visualize protein/DNA complexes. Band intensities were quantified to generate binding curves, and data were fitted to a one-site specific binding model using GraphPad Prism (version 9.0), yielding an apparent $K_{D}$ of 4.06 nM (R^2^ = 0.915).

### 4.7. Surface Plasmon Resonance (SPR)

In DNA-free and DNA-bound conditions, the binding kinetics and affinities between UV-DDB and AAG were determined by SPR on an OpenSPR XT rev4 (Nicoya Lifesciences, Kitchener, ON, Canada; NFEC-2024-09-299613). Experiments were conducted at 25 °C, and all buffers were filtered (0.22 μm) and degassed before use. Carboxyl-functionalized sensor chips were activated using EDC/NHS coupling chemistry, and UV-DDB diluted in sodium acetate buffer (pH 4.5) was immobilized onto the sensor surface, followed by ethanolamine blocking. To reduce nonspecific binding, BSA was applied to both ligand and reference channels, and the reference channel was prepared without UV-DDB immobilization using the same procedure. For DNA-bound conditions, UV-DDB/DNA complexes were assembled using biotinylated THF37 DNA and immobilized on biotin-functionalized sensor surfaces via streptavidin–biotin interactions. After establishing a stable baseline in running buffer, AAG was injected as the analyte at increasing concentrations. The association phase was monitored for 600 s, followed by a 300 s dissociation phase. Sensor surfaces were regenerated using glycine-HCl at an appropriate pH. Data acquisition and processing were performed using TraceDrawer software (version 1.9.1, Nicoya Lifesciences). Raw data were corrected for nonspecific binding via reference subtraction. Reference-subtracted sensorgrams were globally fitted to a 1:1 Langmuir binding model, with the $k_{a}$ and $k_{d}$ constrained as global parameters shared across all analyte concentrations, and the maximum binding capacity ($B_{max}$) treated as a local parameter for each concentration. The $K_{D}$ was calculated as $k_{d}$/$k_{a}$.

### 4.8. Biolayer Interferometry (BLI) Analysis

The binding kinetics and affinities between UV-DDB and AAG, in the absence or presence of DNA, were determined by BLI using an Octet R4 system (Sartorius, Göttingen, Germany). Hydrated AR2G (Amine Reactive Second-Generation, Sartorius, 18-5092) biosensors were activated by immersion in a solution containing 20 mM 1-ethyl-3-(3-dimethylaminopropyl)carbodiimide (EDC) and 10 mM N-hydroxysulfosuccinimide (s-NHS) in a black polypropylene 96-well plate (655209, Greiner Bio-One, Kremsmünster, Austria). UV-DDB (10 μg/mL in sodium acetate, pH 6.0) was subsequently immobilized onto the activated sensors, followed by quenching of the remaining active esters with 1 M ethanolamine (pH 8.5). After establishing a stable baseline in the kinetic buffer (PBS supplemented with 0.1% BSA, 0.02% Tween-20, and 0.05% sodium azide), the sensors were submerged in two distinct analyte preparations: (i) AAG alone (serially diluted from 500 to 4000 nM) and (ii) AAG pre-incubated with 8 nM THF37 dsDNA for 20 min at room temperature. The association phase was monitored for 300 s, followed by a 600 s dissociation phase in the kinetic buffer. Data acquisition and processing were performed using Octet Analysis Studio (version 13.0.0.29, Sartorius) software. Raw data were corrected for non-specific binding by subtracting the signals recorded from a reference sensor (lacking immobilized UV-DDB). Kinetic parameters, including the $k_{a}$, $k_{d}$ and $K_{D}$, were calculated by applying a 1:1 binding model with global fitting.

### 4.9. Structural Prediction of the UV-DDB/AAG Complex Using AlphaFold3

The three-dimensional structures of the UV-DDB/AAG complex were predicted using the AlphaFold3 web server (https://alphafoldserver.com), which employs a diffusion-based deep learning architecture for modeling interactions among proteins, nucleic acids, and other biomolecules [28]. Predictions were performed under two conditions: in the absence of DNA and in the presence of an AP site-containing dsDNA substrate.

For DNA-free predictions, three protein components were submitted as separate entities: human DDB1 (UniProt accession Q16531), human DDB2 (UniProt accession Q92466), and human AAG (UniProt accession P29372). For DNA-bound predictions, the same protein components were included together with a double-stranded DNA substrate corresponding to the THF37 AP site sequence used in biochemical assays. The top strand (5′-CCG AGT CAT TCC TGC AGC GXG TCC ATG GGA GTC AAA T-3′, where X denotes a THF AP site analog) was submitted, with the complementary strand automatically generated using the reverse complement function of the AlphaFold3 server. All three proteins were submitted as full-length sequences corresponding to their respective UniProt entries. Each prediction was performed using default server parameters, generating five candidate models per run. The highest-ranked model was selected based on pTM (predicted template modeling score) and ipTM (interface predicted template modeling score) and used for subsequent analyses. pTM, ipTM, and PAE (predicted aligned error) plots for the selected models are provided in the Supplementary Information (Supplementary Figures S3A–D and S4).

Predicted structures were visualized and analyzed using PyMOL (version 3.1.8, Schrödinger, New York, NY, USA). Per-residue pLDDT values were examined to assess local structural confidence. Residues with pLDDT (predicted local distance difference test) < 50 were considered structurally disordered and excluded from interface analysis and structural visualization. Specifically, the N-terminal regions of DDB2 (residues 1–64) and AAG (residues 1–83) were excluded from visualization. At the protein/protein interface in the DNA-free model, putative contact residues were identified using an interatomic distance threshold of 3.5 Å. For the DNA-bound ternary complex, distances between AAG active site residues and the C1′ atom of the THF AP site residue were measured based on residues with established functional roles in prior structural studies. Structural comparisons between DNA-free and DNA-bound models were performed using pairwise alignment in PyMOL (version 3.1.8, Schrödinger) to identify potential DNA-dependent conformational rearrangements at the UV-DDB/AAG interface [28].

## Figures and Tables

**Table 1:** Predicted contact residues at the DDB1/AAG interface in the absence of DNA, based on AlphaFold3 structural modeling.

Residue (AAG)	Atom	Residue (DDB1)	Atom	Distance (Å)	Predicted Interaction Type
Arg145	NE	Glu800	OE2	2.1	H-bond; salt bridge
Arg145	NH1	Thr798	OG1	3.4	H-bond
Arg145	NH2	Glu800	OE2	2.9	H-bond; salt bridge
Gln195	NE2	Glu342	OE2	3.4	H-bond
Lys229	NZ	Gln759	O	3.3	H-bond
Lys229	NZ	Glu800	OE1	2.9	H-bond; salt bridge

Distances were measured between the indicated atoms in the AlphaFold3-predicted structure.

**Table 2:** Distance comparison of wild-type DDB1/AAG interface contacts in alanine substitution models, based on AlphaFold3 structural predictions.

Residue (DDB1)	Atom	Residue (AAG)	Atom	WT (Å)	Glu800Ala (Å)	Arg145Ala (Å)	Lys229Ala (Å)
Glu800	OE2	Arg145	NE	2.1	40.1	41.4	32.1
Glu800	OE2	Arg145	NH2	2.9	38.5	41.4	31.7
Glu800	OE1	Lys229	NZ	2.9	40.4	34.5	25.1
Thr798	OG1	Arg145	NH1	3.4	37.9	42.3	33.5
Glu342	OE2	Gln195	NE2	3.4	43.5	13.0	18.7
Gln759	O	Lys229	NZ	3.3	47.1	40.8	30.0

**Table 3:** Predicted contact residues at the DDB2/DNA and AAG/DNA interfaces in the UV-DDB/DNA/AAG ternary complex, based on AlphaFold3 structural modeling.

Protein	Residue	Atom	Target	Distance (Å)	Putative Functional Role	Literature Basis
AAG	Tyr127	OH	AP site (C1′)	2.4	Stacking interaction with flipped-out base; essential invariant residue	[29]
	Leu180	CD2	AP site (C1′)	3.3	Substrate discrimination	[30]
	Glu125	OE2	AP site (C1′)	3.7	Catalytic residue; general base for water activation and N-glycosidic bond cleavage	[29]
	Tyr159	OH	AP site (C1′)	4.2	Base orientation; perpendicular stacking with Tyr127	[29]
	Arg182	NH1	AP site (C1′)	4.8	Water molecule coordination; cooperates with Glu125	[31]
	His136	NE2	AP site (C1′)	6.6	Hydrogen bond with flipped-out base; essential invariant residue	[29]
	Tyr162	OH	AP site (C1′)	9.1	Intercalating residue; stabilizes extrahelical conformation during nucleotide flipping	[29,32,33]

Distances were measured between the indicated atoms and the THF abasic site residue (position 20) in the AlphaFold3-predicted ternary complex structure.

## Data Availability

The AlphaFold3-predicted structures of the AAG/UV-DDB complex generated in this study are openly available in Zenodo at https://doi.org/10.5281/zenodo.20175879. The raw EMSA and SPR data supporting the conclusions of this article will be made available by the authors on request.

## Supplementary Materials

The following supporting information can be downloaded at https://www.mdpi.com/article/10.3390/ijms27125521/s1.

## Abbreviations

BER	base excision repair
NER	nucleotide excision repair
UV	ultraviolet
UV-DDB	ultraviolet-damaged DNA-binding protein
AAG	alkyladenine DNA glycosylase
EMSA	electrophoretic mobility shift assay
SPR	surface plasmon resonance
BLI	biolayer interferometry
$K_{D}$	equilibrium dissociation constant
AP site	apurinic/apyrimidinic site
DDB1	DNA damage-binding protein 1
DDB2	DNA damage-binding protein 2
CUL4A/RBX1	Cullin 4A/RING-box protein 1
PARP-1	poly(ADP-ribose) polymerase-1
MutM	formamidopyrimidine-DNA glycosylase
MutS	DNA mismatch repair protein MutS
RPA	replication protein A
APE1	apurinic/apyrimidinic endonuclease 1
FLAG	FLAG epitope tag
HA	hemagglutinin tag
SDS-PAGE	sodium dodecyl sulfate–polyacrylamide gel electrophoresis
SEC	size-exclusion chromatography
THF	tetrahydrofuran
IRDye 800CW	infrared dye 800, cyanine-based water-soluble dye
$B_{max}$	maximum binding capacity
$k_{a}$	association rate constant
$k_{d}$	dissociation rate constant
Arg	arginine
Gln	glutamine
Lys	lysine
Glu	glutamic acid
Thr	threonine
Tyr	tyrosine
Leu	leucine
His	histidine
NZ	nitrogen atom of lysine side chain
XPE	xeroderma pigmentosum group E
DCAF	DDB1- and cullin 4-associated factor
XPF	xeroderma pigmentosum complementation group F
ERCC1	excision repair cross-complementation group 1
OGG1	8-oxoguanine DNA glycosylase 1
MUTYH	MutY DNA glycosylase homolog
SMUG1	single-strand-selective monofunctional uracil-DNA glycosylase 1
XL-MS	crosslinking mass spectrometry
cryo-EM	cryo-electron microscopy
εA	1,N^6^-ethenoadenine
EDTA	ethylenediaminetetraacetic acid
TCEP	tris(2-carboxyethyl)phosphine
PBS	phosphate-buffered saline
HEPES	4-(2-hydroxyethyl)-1-piperazineethanesulfonic acid
DTT	dithiothreitol
OD_600_	optical density at 600 nm
IPTG	isopropyl β-D-1-thiogalactopyranoside
PMSF	phenylmethylsulfonyl fluoride
BCA	bicinchoninic acid
PVDF	polyvinylidene fluoride
HRP	horseradish peroxidase
ECL	enhanced chemiluminescence
HPLC	high-performance liquid chromatography
BSA	bovine serum albumin
TBE	tris-borate-ethylenediaminetetraacetic acid
EDC	1-ethyl-3-(3-dimethylaminopropyl)carbodiimide
NHS	N-hydroxysuccinimide
s-NHS	N-hydroxysulfosuccinimide
AR2G	Amine Reactive Second-Generation biosensor
pTM	predicted template modeling score
ipTM	interface predicted template modeling score
PAE	predicted aligned error
pLDDT	predicted local distance difference test
