# Supplementary material for: UV-DDB as a Dynamic Regulator Linking Base Excision and Nucleotide Excision Repair via AAG Interaction
Source: Int J Mol Sci. 2026 Jun 18;27(12):5521. doi: 10.3390/ijms27125521 (PMC13299579; doi:10.3390/ijms27125521)
Supplement: Supplementary file 1 [file ijms-27-05521-s001.zip › ijms-4299420-supplementary.pdf]

Supplementary Information

A

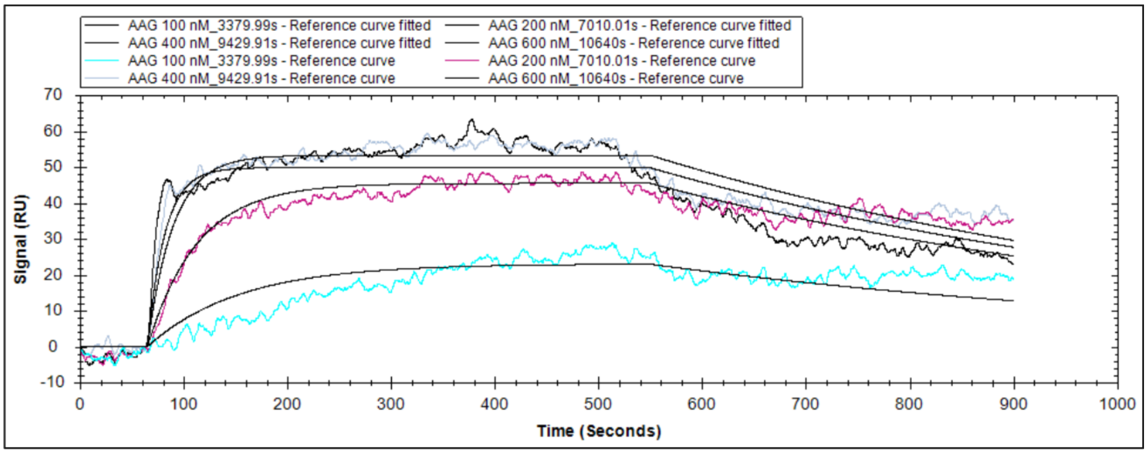

B

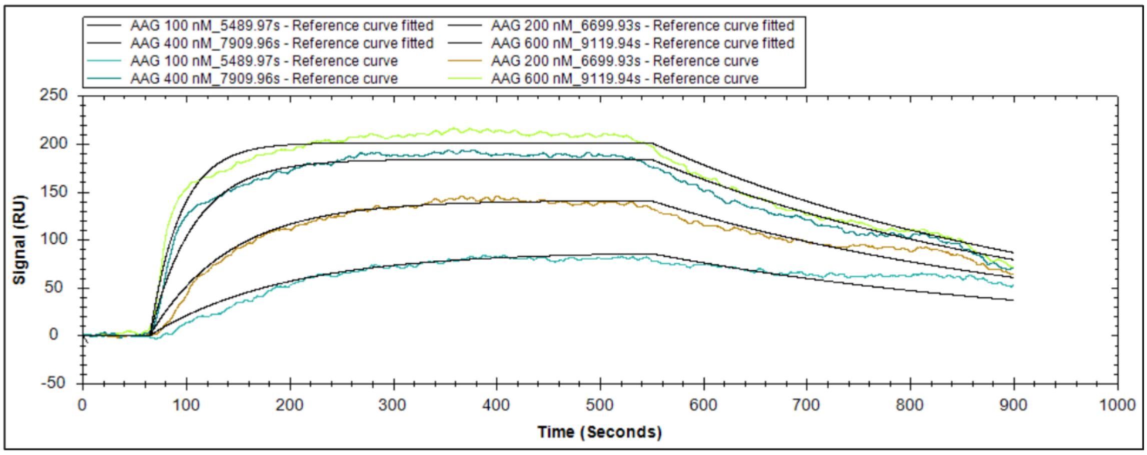

Supplementary Figure S1. Representative SPR sensorgrams with globally fitted curves.

(A) Representative overlay of experimental sensorgrams (colored curves) and globally fitted curves (black lines) for AAG binding to immobilized UV-DDB in the absence of DNA.

(B) Representative overlay of experimental sensorgrams (colored curves) and globally fitted curves (black lines) for AAG binding to immobilized UV-DDB in the presence of AP site-containing DNA.

Parameter	Absence of DNA ± SE	Presence of AP site-containing DNA ± SE
k_a ($M^{-1}s^{-1}$)	$9.58 \times 10^4 \pm 5.14 \times 10^2$	$5.22 \times 10^4 \pm 1.55 \times 10^2$
k_d (s^{-1})	$1.68 \times 10^{-3} \pm 1.99 \times 10^{-6}$	$2.41 \times 10^{-3} \pm 1.57 \times 10^{-5}$
K_D (M)	$1.75 \times 10^{-8} \pm 1.15 \times 10^{-10}$	$4.62 \times 10^{-8} \pm 4.39 \times 10^{-10}$

Supplementary Table S1. Kinetic parameters with standard errors (SE) from global fitting of representative SPR sensorgrams for AAG binding to immobilized UV-DDB in the absence and presence of AP site-containing DNA.

Standard errors (SE) were obtained from global fitting using TraceDrawer software (Nicoya Lifesciences).

Parameter	Experiment 1	Experiment 2	Experiment 3	Mean ± SD
k_a ($M^{-1}s^{-1}$)	3.53×10^4	9.58×10^4	2.48×10^5	$(1.26 \pm 1.10) \times 10^5$
k_d (s^{-1})	1.91×10^{-3}	1.68×10^{-3}	1.16×10^{-3}	$(1.58 \pm 0.38) \times 10^{-3}$
K_D (M)	5.43×10^{-8}	1.75×10^{-8}	4.66×10^{-9}	$(2.55 \pm 2.58) \times 10^{-8}$

Supplementary Table S2A. Kinetic parameters from replicate SPR experiments for AAG binding to immobilized UV-DDB in the absence of DNA (n = 3).

Parameter	Experiment 1	Experiment 2	Experiment 3	Mean ± SD
k_a ($M^{-1}s^{-1}$)	5.22×10^4	2.23×10^4	3.53×10^4	$(3.66 \pm 1.51) \times 10^4$
k_d (s^{-1})	2.41×10^{-3}	1.99×10^{-3}	2.53×10^{-3}	$(2.31 \pm 0.28) \times 10^{-3}$
K_D (M)	4.62×10^{-8}	8.93×10^{-8}	7.16×10^{-8}	$(6.90 \pm 2.17) \times 10^{-8}$

Supplementary Table S2B. Kinetic parameters from replicate SPR experiments for AAG binding to immobilized UV-DDB/AP site-containing DNA complexes (n = 3).

Mean ± SD values in Supplementary Tables S2A and S2B were calculated from three experiments.

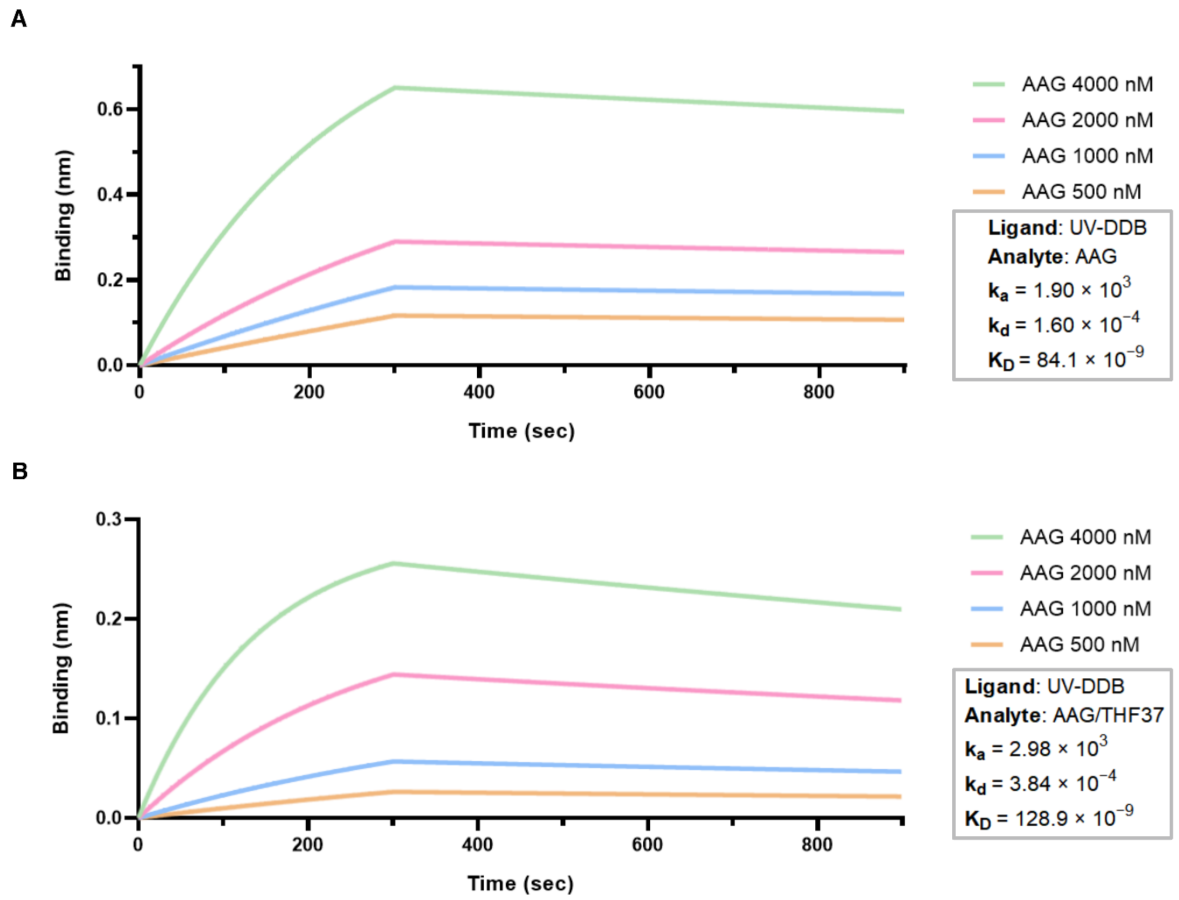

Supplementary Figure S2. BLI analysis of AAG binding to UV-DDB in the absence and presence of DNA.

(A) Sensorgrams showing concentration-dependent binding of AAG to immobilized UV-DDB in the absence of DNA. The binding signal increased with AAG concentration during the association phase and approached saturation at higher concentrations. During the dissociation phase, limited signal decay was observed, indicating slow dissociation kinetics and formation of a stable complex. Data were globally fitted to a 1:1 binding model, yielding a dissociation constant (K_D) of approximately 84.1 nM (R^2 = 0.9892).

(B) Sensorgrams showing concentration-dependent binding of AAG pre-incubated with THF37 dsDNA to immobilized UV-DDB. The binding signal increased with AAG concentration during the association phase and approached saturation at higher concentrations. During the dissociation phase, limited signal decay was observed, indicating slow dissociation kinetics and persistence of the complex. Data were globally fitted to a 1:1 binding model, yielding a dissociation constant (K_D) of approximately 128.9 nM (R^2 = 0.9375).

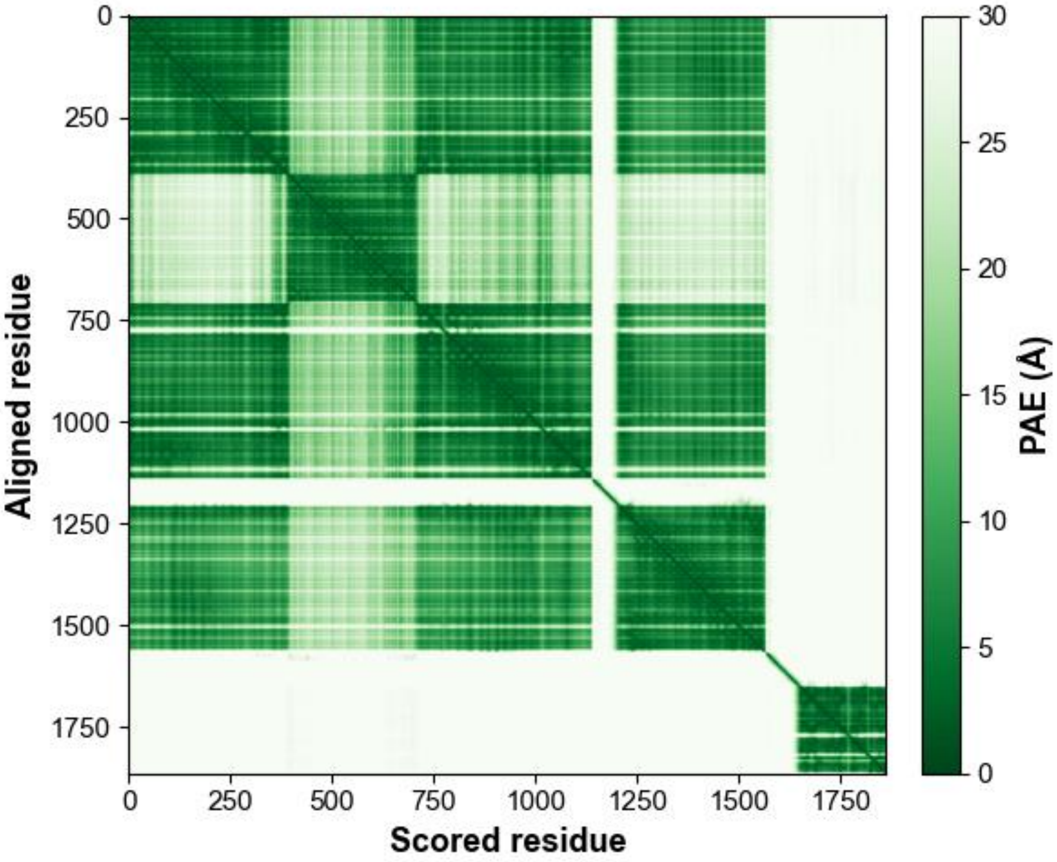

Supplementary Figure S3A. Predicted Aligned Error (PAE) plot of the AlphaFold3-predicted UV-DDB/AAG complex in the absence of DNA.

ipTM: 0.69, pTM: 0.71

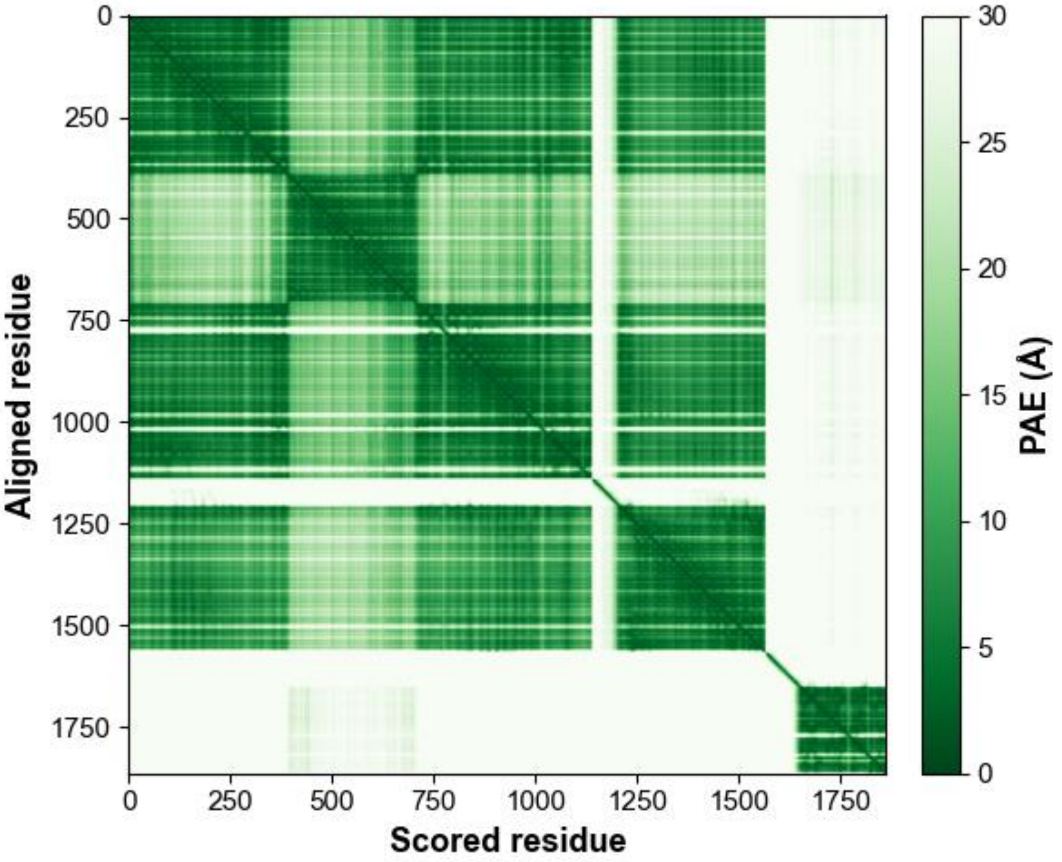

Supplementary Figure S3B. PAE plot of the AlphaFold3-predicted UV-DDB(DDB1 Glu800Ala)/AAG complex in the absence of DNA.

ipTM = 0.7, pTM = 0.72

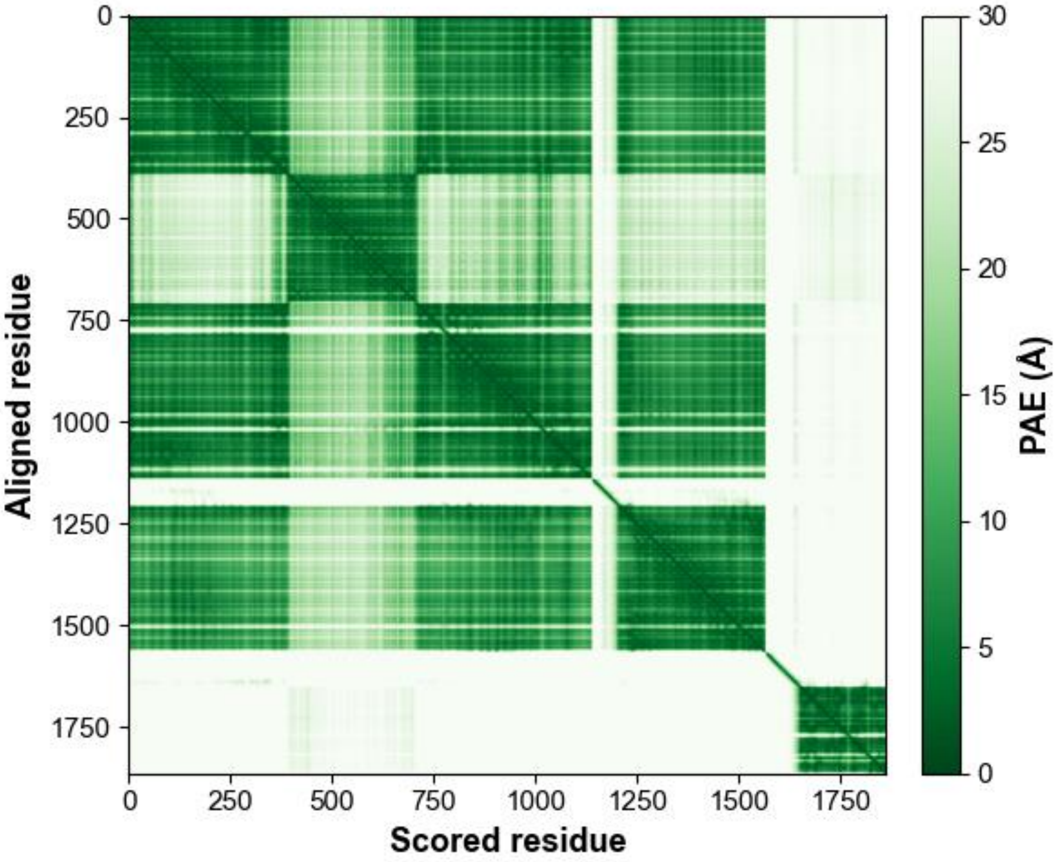

Supplementary Figure S3C. PAE plot of the AlphaFold3-predicted UV-DDB/AAG(Arg145Ala) complex in the absence of DNA.

ipTM = 0.68, pTM = 0.71

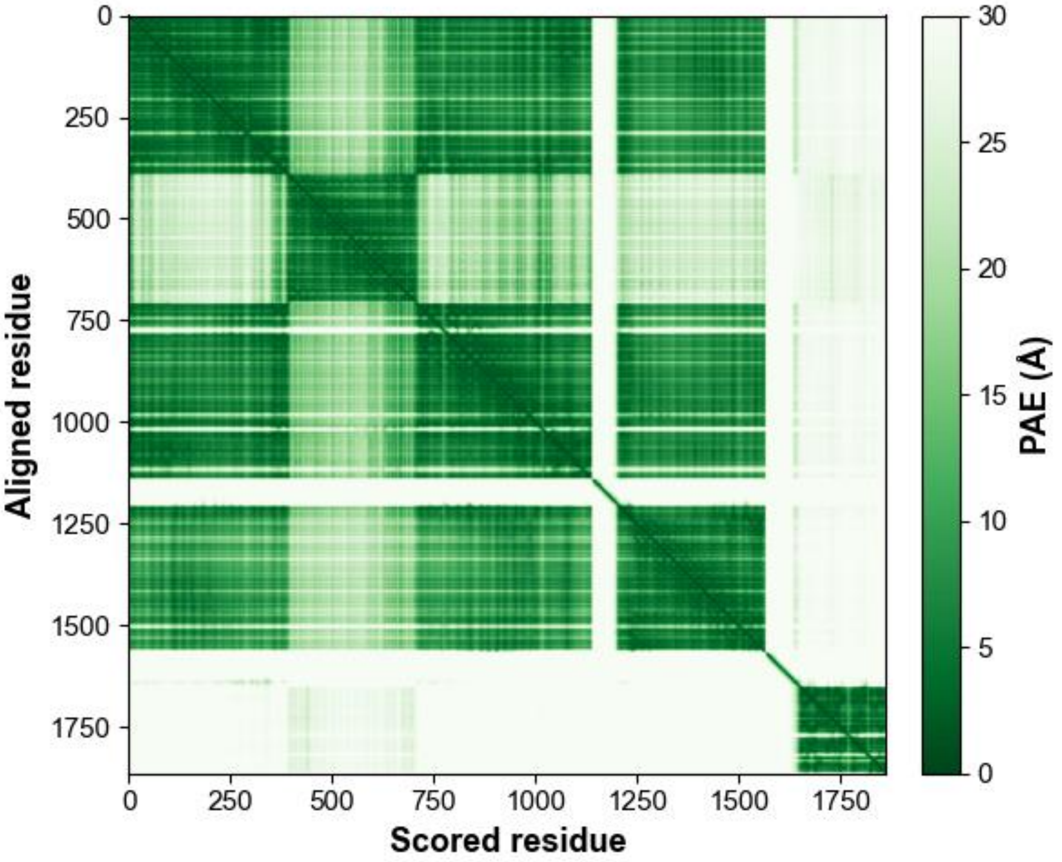

Supplementary Figure S3D. PAE plot of the AlphaFold3-predicted UV-DDB/AAG(Lys229Ala) complex in the absence of DNA.

ipTM = 0.69, pTM = 0.72

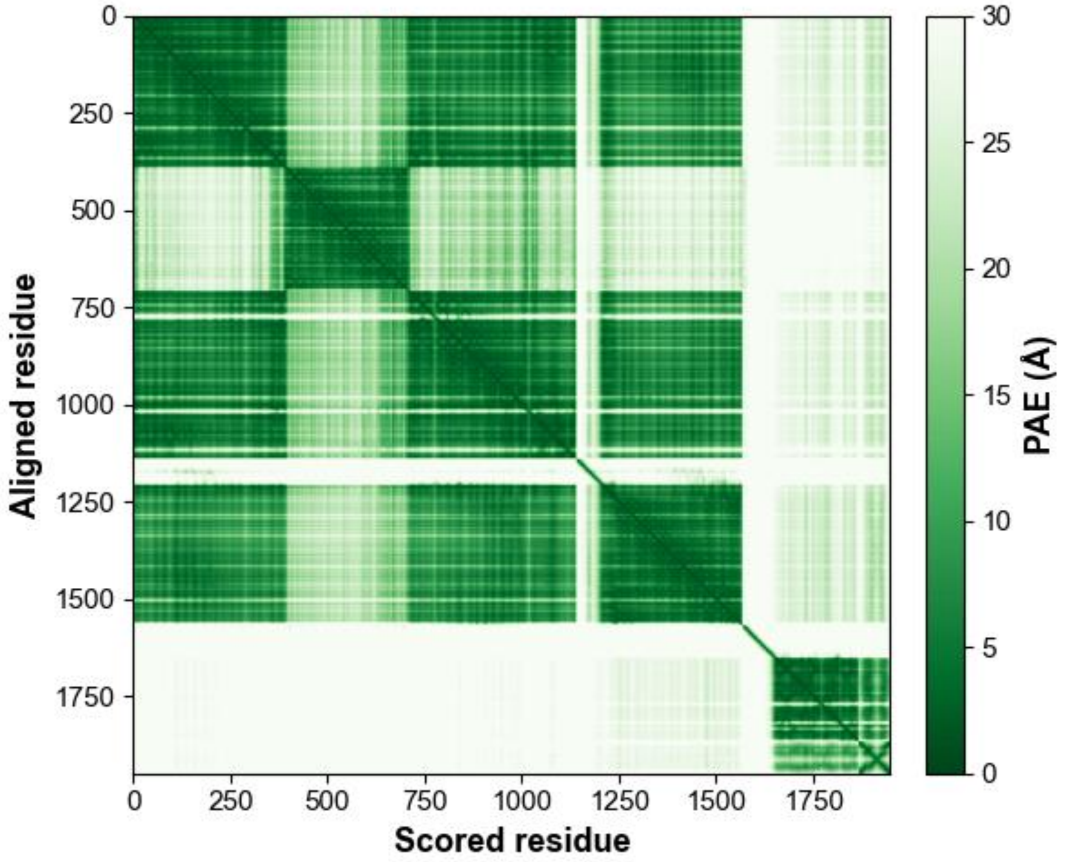

Supplementary Figure S4. PAE plot of the AlphaFold3-predicted UV-DDB/DNA/AAG ternary complex.

ipTM = 0.69, pTM = 0.71
